# Genomic and metagenomic analysis of microbes in a soil environment affected by the 2011 Great East Japan Earthquake tsunami

**DOI:** 10.1186/s12864-016-2380-4

**Published:** 2016-01-14

**Authors:** Satoshi Hiraoka, Asako Machiyama, Minoru Ijichi, Kentaro Inoue, Kenshiro Oshima, Masahira Hattori, Susumu Yoshizawa, Kazuhiro Kogure, Wataru Iwasaki

**Affiliations:** Department of Computational Biology and Medical Sciences, Graduate School of Frontier Sciences, the University of Tokyo, Chiba, 277-8568 Japan; Department of Biological Sciences, Graduate School of Science, the University of Tokyo, Tokyo, 113-0032 Japan; Atmosphere and Ocean Research Institute, the University of Tokyo, Chiba, 277-8564 Japan; Center for Omics and Bioinformatics, Graduate School of Frontier Sciences, The University of Tokyo, Chiba, 277–8561 Japan

**Keywords:** Arthrobacter, Comparative genomics, Environmental microbes, Metagenomics, Siderophore, Tsunami

## Abstract

**Background:**

The Great East Japan Earthquake of 2011 triggered large tsunami waves, which flooded broad areas of land along the Pacific coast of eastern Japan and changed the soil environment drastically. However, the microbial characteristics of tsunami-affected soil at the genomic level remain largely unknown. In this study, we isolated microbes from a soil sample using general low-nutrient and seawater-based media to investigate microbial characteristics in tsunami-affected soil.

**Results:**

As expected, a greater proportion of strains isolated from the tsunami-affected soil than the unaffected soil grew in the seawater-based medium. Cultivable strains in both the general low-nutrient and seawater-based media were distributed in the genus *Arthrobacter*. Most importantly, whole-genome sequencing of four of the isolated *Arthrobacter* strains revealed independent losses of siderophore-synthesis genes from their genomes. Siderophores are low-molecular-weight, iron-chelating compounds that are secreted for iron uptake; thus, the loss of siderophore-synthesis genes indicates that these strains have adapted to environments with high-iron concentrations. Indeed, chemical analysis confirmed the investigated soil samples to be rich in iron, and culture experiments confirmed weak cultivability of some of these strains in iron-limited media. Furthermore, metagenomic analyses demonstrated over-representation of denitrification-related genes in the tsunami-affected soil sample, as well as the presence of pathogenic and marine-living genera and genes related to salt-tolerance.

**Conclusions:**

Collectively, the present results would provide an example of microbial characteristics of soil disturbed by the tsunami, which may give an insight into microbial adaptation to drastic environmental changes. Further analyses on microbial ecology after a tsunami are envisioned to develop a deeper understanding of the recovery processes of terrestrial microbial ecosystems.

**Electronic supplementary material:**

The online version of this article (doi:10.1186/s12864-016-2380-4) contains supplementary material, which is available to authorized users.

## Background

On March 11, 2011, the Great East Japan Earthquake occurred off the coast of Tohoku, Japan. The earthquake triggered large tsunami waves, which flooded broad areas of land along the Pacific coast and changed the soil environment due to seawater and sludge that originated from marine sediments [1]. Previous studies showed that following the Indian Ocean tsunami of December 26, 2004, the tsunami-affected areas maintained high-salinity conditions for over eight months [2], and there were also changes in several chemical characteristics, including an increase in organic matter content [3], increase in nitrate and phosphate content [4], increase in heavy-metal ion concentrations [5–7], decrease in pH, and increase in electrical conductivity [8]. Increases in salinity and organic matter were also reported at a number of places along the pacific coast following the Tohoku tsunami [1].

Such changes in the soil environment after the tsunami are also likely to have an impact on the ecosystem. There have been many studies conducted to date investigating how such changes affect plants; for example, vegetation senescence was reported after the Indian Ocean tsunami [7, 9, 10] and flora variations on sandy beaches were observed after the Tohoku tsunami [11]. On the other hand, only a few studies have evaluated the effects of a tsunami on microbes. Somboonna et al. applied 16S ribosomal RNA (rRNA) amplicon sequencing to the soil affected by the Indian Ocean tsunami and observed changes in the microbial population structure [12]. Wada et al. also used 16S rRNA amplicon sequencing to analyze samples of the sludge brought ashore by the Tohoku tsunami and identified several pathogenic and sulfate-reducing bacterial groups [13]. However, no study has yet investigated the microbial characteristics of tsunami-affected soil at the genomic level.

In this study, we evaluated the microbial characteristics of a soil environment affected by the Tohoku tsunami, using whole-genome and shotgun metagenome sequencing approaches. Notably, whole-genome sequencing of four *Arthrobacter* strains isolated from the tsunami-affected soil sample revealed that siderophore-synthesis genes were independently lost in each genome. Siderophores are compounds that function in iron absorption [14–16], and these gene losses were consistent with the results of soil chemical analysis and culture experiments under iron-controlled conditions. Furthermore, metagenomic analyses indicated over-representation of denitrification-related genes in the tsunami-affected soil sample, as well as the existence of pathogenic and marine-living genera and genes related to salt-tolerance.

## Materials and methods

### Sample collection

**Fig. 1:**
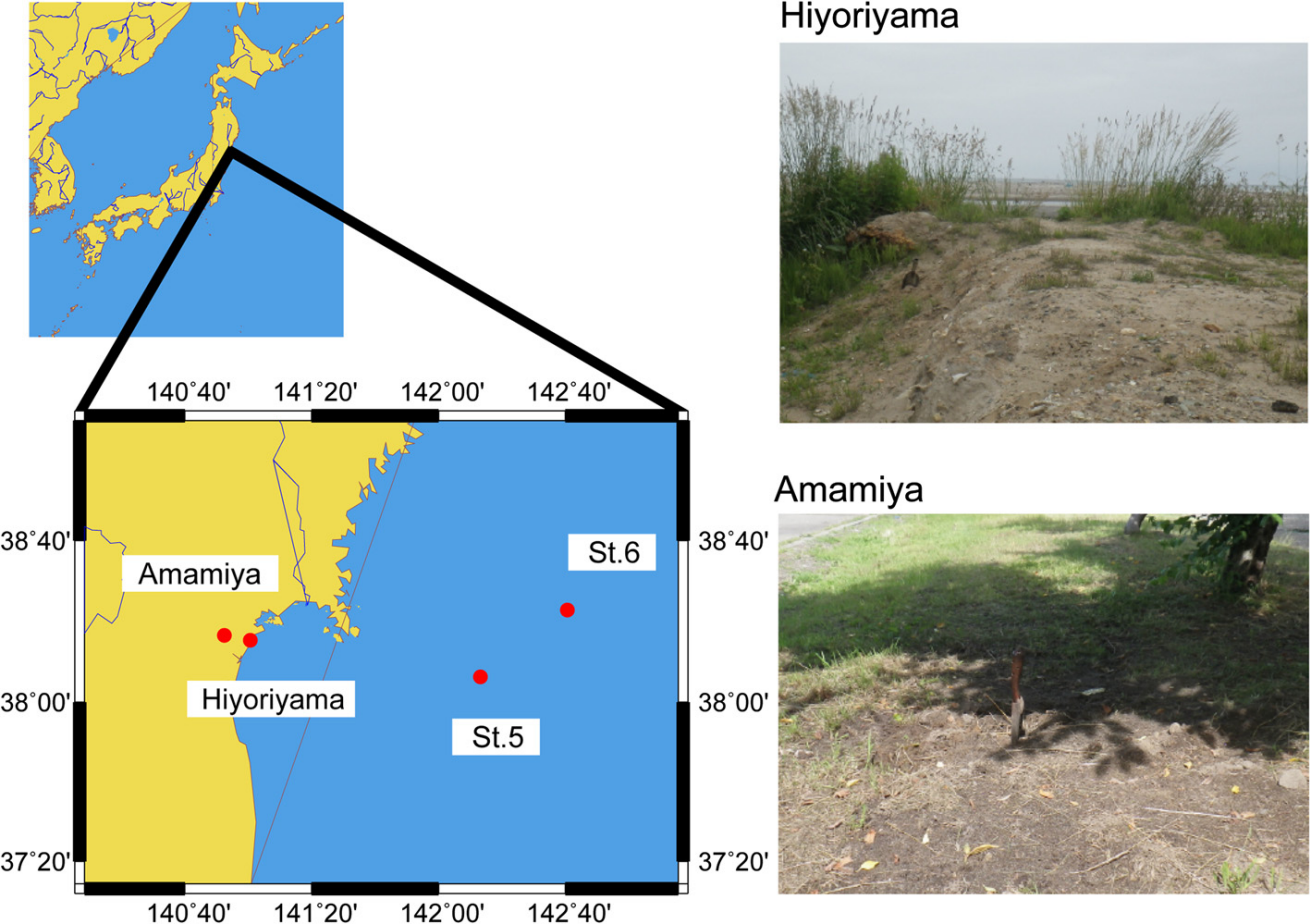
A map and photos of the sampling sites in a coastal area of Sendai, Japan. The Tohoku tsunami reached Hiyoriyama, but not Amamiya

Soil samplings were conducted at Hiyoriyama (38°15’20”N, 141°0’42”E) and Amamiya (38°16’35”N, 140°52’16”E) in Sendai city, Miyagi, Japan, in July 2012 (Fig. 1). If needed, the owners of the lands gave permission to conduct the study on these sites. We confirm that the study did not involve endangered or protected species. The Hiyoriyama site is 0.5 km off the coastline and was affected by the tsunami, whereas the Amamiya site is 12 km off the coastline and was not affected; the two sites are 13 km apart. The surface soil was removed to a 5 cm depth before sampling. Intermingled plants were carefully removed using tweezers, and soil that passed through a 2.0-mm pore-sized sieve was collected. The collected soil samples were transported to the laboratory at 4 °C and immediately stored at -80 °C until the subsequent analysis.

Seawater sampling was conducted at St.5 (38°06’00”N, 142°15’00”E) and St.6 (38°22’59”N, 142°43’01”E) off the coast of Miyagi, Japan in the Pacific Ocean, in August 2012, during the KT-12-21 cruise of R/V Tansei-Maru (JURCAOS, JAMSTEC). The St.5 and St.6 stations are located 110 km and 150 km from Sendai city, respectively. Surface seawater was collected in a prewashed bucket and immediately spread onto agar plates on a research vessel.

### Isolation and 16S rRNA sequencing

R2A medium (Wako Pure Chemical Industries) was used to cultivate microbial strains that grow under general, low-nutrient condition, and ZoBell marine medium (Becton Dickinson and Company) was used to cultivate microbial strains that have adapted to a seawater-affected condition. Soil samples were thawed at 4 °C overnight, suspended in R2A or ZoBell liquid medium, and plated to the corresponding agar medium at a density of 10 ^−4^ g soil per plate with five replicates. The plates were incubated at 20 °C for 7 days before colony counting and picking. To obtain strains that could grow in both media, single colonies on the R2A agar plates were transferred to ZoBell agar plates with sterilized sticks, incubated at 20 °C for 7 days, and isolated by spread-plating on ZoBell agar at 20 °C. The seawater samples were plated to R2A and ZoBell agar at a volume of 100 *μ*L seawater per plate with three replicates. The plates were incubated at 20 °C for 7 days before colony counting and picking.

To sequence 16S rRNA genes, seven strains that were isolated from the Hiyoriyama site and could actively grow in both R2A and ZoBell media were randomly selected. After incubation in ZoBell liquid medium, DNA was extracted using Wizard Genomic DNA Purification Kit (Promega). The 16S rRNA genes were amplified using a standard polymerase chain reaction protocol with the primers 27F (5’-AGAGTTTGATCMTGGCTCAG-3’) and 1492R (5’-GGCTACCTTGTTACGACTT-3’) [17], and sequenced by the Sanger method.

### Whole-genome sequencing and analysis

Four strains of the *Arthrobacter* genus that were isolated from the Hiyoriyama site and were cultivable in both R2A and ZoBell media were targeted for whole-genome sequencing. Genomic DNA was extracted by the phenol-chloroform method. Two strains (named Hiyo4 and Hiyo8) were sequenced using PacBio RS II (Pacific Biosciences) according to the manufacturer protocols. *De novo* genome assembly of the 62,608 (Hiyo4) and 65,240 (Hiyo8) raw reads obtained from the Sprai pipeline (http://zombie.cb.k.u-tokyo.ac.jp/sprai/) successfully generated one and three circular contigs, respectively, after manual curation. The other two strains (Hiyo1 and Hiyo6) were sequenced using GS FLX+ System (Roche) and Ion PGM (Thermo Fisher Scientific) according to the manufacturer protocols. *De novo* genome assembly of the 301,881 (Hiyo1) and 267,295 (Hiyo6) reads obtained from the Newbler assembler [18] generated 38 and 630 scaffolds, respectively.

Coding sequences (CDSs) were predicted by applying Prodigal [19] to the contig sequences. Functional annotation was performed by blastp searches [20] against the Swiss-Prot [21] and eggNOG v4.0 [22] databases with a cut-off e-value ≤1E-5. Transfer RNA (tRNA) and rRNA sequences were predicted using tRNAscan-SE [23] and RNAmmer [24], respectively, with default settings.

For comparative genome analysis, all 21 publicly available genome sequences (6 complete and 15 draft sequences) of the *Arthrobacter* genus were downloaded from GenBank [25] via EzGenome (http://www.ezbiocloud.net/ezgenome) in January 2015 (Additional file 1). The CDSs of the four isolated and 21 downloaded genomes were subjected to blastp searches against the eggNOG database [22] with cut-off e-value ≤1E-5 and identity ≥90 %.

For construction of a phylogenetic tree, the 16S rRNA sequences of 56 *Arthrobacter* type strains and *Streptomyces coelicoflavus* NBRC 15399^T^ were additionally downloaded from the RDP webserver [26]. *Streptomyces coelicoflavus* NBRC 15399^T^ was used as an outgroup [27]. The 16S rRNA sequences of the total 82 strains were subjected to multiple alignment using MUSCLE [28] with default settings. A maximum-likelihood (ML) tree was generated by MEGA 6 [29] with the K80 substitution model with a gamma distribution and invariant sites (K2+G+I), which was the AIC-selected model, and 1000 bootstrap replicates. An ML tree of the total 17 genome-available strains was constructed on the basis of the set of 400 conserved bacterial marker genes using PhyloPhlAn [30] and MEGA 6 [29] with the WAG substitution model that incorporates gamma distribution and the amino-acid frequencies of the dataset (WAG+G+F), which was the AIC-selected model, and 1000 bootstrap replicates.

### Culture assays of iron dependency

To determine the difference in iron tolerance among strains in relation to the genetic analysis results, culture assays were conducted at different iron concentrations. In addition to the four isolated *Arthrobacter* strains, we cultivated four closely related and genome-sequenced species, *A. aurescens* Phillips 1953^T^ (JCM 1330^T^), *A. chlorophenolicus* A6^T^ (JCM 12360^T^), *A. globiformis* Conn 1928^T^ (JCM 1332^T^), and *A. phenanthrenivorans* Sphe3^T^ (JCM 16027^T^). All four species had intact siderophore-synthesis genes in their genomes. These strains were provided by the Japan Collection of Microorganisms, BioResource Center, RIKEN and National BioResource Project of Ministry of Education, Culture, Sports, Science and Technology, Japan.

Iron-controlled, modified MM9 medium was prepared as follows. A solution containing 0.3 g/L KH_2_PO_4_, 0.5 g/L NaCl, 1.0 g/L NH_4_Cl, 6.0 g/L NaOH, and 30.24 g/L PIPES was adjusted to pH 7.0 with NaOH. After autoclaving, separately sterilized solutions of 10 mL of 20 wt% glucose, 1 mL of 1 M MgCl_2_, and 0.1 mL of 1 M CaCl_2_ were added to 1 L of the solution [31]. Then, the iron concentration was adjusted to 0, 0.1, 1, or 10 *μ*M with a FeCl_3_-containing solution that was prepared in the same manner.

Each strain was precultured until its optical density at 660 nm (OD_660_) reached 0.1 in the iron-free modified MM9 liquid medium. Then, 100 *μ*L of the suspension was inoculated to 50-mL tubes containing 10 mL of the iron-controlled, modified MM9 medium. Among the additional four strains, only *A. phenanthrenivorans* Sphe3 showed growth in the modified MM9 medium. The tubes were incubated at 30 °C on a linear shaker at 200 rpm for 3 days, and the OD_660_ was measured periodically during the incubation period. The growth curve was fitted to the logistic model to calculate the maximum growth rate.

### Soil chemical analysis

The soil samples were subjected to chemical analysis for pH, electrical conductivity, and concentrations of total organic carbon, total nitrogen, nitrate, nitrite, ammonium, effective phosphate, exchangeable ions (K^+^, Ca^2+^, Mg^2+^, Na^+^, and Mn^2+^), available iron (Fe), chloride ion (Cl^-^), sulfate ion (SO$_{4}^{2-}$), eluted heavy metals (Cd, Cr (VI), total Hg, alkyl mercury, Pb, As, and B), and contained heavy metals (Cd, Cr (VI), Hg, Pb, As, B, Cu, Zn, and Ni). The analysis was conducted by Createrra Inc. (Tokyo, Japan).

### Shotgun metagenome sequencing and analysis

Metagenomic DNA was extracted using PowerSoil DNA Isolation Kit (MoBio Laboratories). Shotgun metagenome sequencing was performed using the GS FLX+ System according to the supplier’s protocol. Duplicated reads were removed by CD-HIT-454 [32].

Taxonomic assignment was performed using Kraken [33] against complete prokaryotic genomes from RefSeq [34]. CDS prediction was performed using MetaProdigal [35]. CDSs less than 30 amino acids in length were excluded from further analysis. Functional annotations were based on blastp searches against the eggNOG [22] and Swiss-Prot [21] databases with a cut-off e-value ≤1E-5.

SortMeRNA [36] was applied to the shotgun metagenome data to extract 16S rRNA sequences. For each extracted 16S rRNA sequence, a blastn search was performed against MetaMetaDB [37] and the top hit sequences with e-value ≤1E-10 and identity ≥90 % were retrieved. Microbial habitability index (MHI) scores were calculated as described previously [37].

### Data deposition

The whole-genome and plasmid sequence data of Hiyo1, Hiyo4, Hiyo6, and Hiyo8 were deposited in the DDBJ/ENA/GenBank database under the BioSample ID SAMD00024042, SAMD00024043, SAMD00024044, and SAMD00024045, respectively. The shotgun metagenome sequence data of Hiyoriyama and Amamiya were deposited in the DDBJ/ENA/GenBank database under BioSample ID SAMD00023516 and SAMD00023517, respectively. All data were registered under BioProject ID PRJDB3373.

## Results and discussion

### Isolation of microbial strains

To investigate whether the microbial community at the Hiyoriyama (tsunami-affected) site contained more microbes that are adapted to seawater-affected conditions than that at the Amamiya (tsunami-unaffected) site, we conducted culture experiments using R2A (general low-nutrient) and ZoBell (seawater-based) media. At Hiyoriyama, the mean (± standard deviation) numbers of colony forming unit (CFU) per gram of soil were 7.0 ± 3.9 ×10^5^ and 3.0 ± 2.0 ×10^5^ on R2A and ZoBell, respectively. At Amamiya, these numbers of CFU were 21.8 ± 4.7 ×10^5^ and 3.6 ± 2.3 ×10^5^. The ZoBell/R2A CFU ratios were 0.43 and 0.17 at Hiyoriyama and Amamiya, respectively, indicating that the Hiyoriyama site would be comparatively enriched with microbes adapted to a seawater-affected condition at 10 months after the tsunami. For comparison, surface seawater samples collected at St. 5 and St. 6 in the offshore were spread onto both agar plates. The numbers of CFU per milliliter of seawater were 12.7 ± 13.3 ×10^1^ and 81.7 ± 43.6 ×10^1^ on R2A and ZoBell, respectively. As expected, the ZoBell/R2A CFU ratio (6.4) was significantly higher at the offshore sites than at Amamiya and Hiyoriyama (p-value <0.05, t-test).

To isolate microbial strains that are potentially adapted to both types of environments from Hiyoriyama, we aseptically transferred the microbial colonies grown on R2A to ZoBell agar plates. Seven isolated colonies were randomly picked up and their 16S rRNA genes were sequenced. Unexpectedly, all of the strains were found to belong to a single genus, *Arthrobacter*. The genus *Arthrobacter* is an aerobic, gram-positive member of the family *Micrococcaceae*, *Actinobacteria* [38, 39]. This genus is broadly found in soils, as well as in extreme environments, including the deep subsurface [40], arctic ice [41], radioactive sites [42], and heavy metal-contaminated sites [43]. Some *Arthrobacter* species were reported to tolerate drastic environmental stresses, e.g., desiccation [44], starvation [45], heavy metals [46, 47], and radioactivity [48]. Furthermore, at the time of analysis, 6 complete and 15 draft genome sequences were available for comparative genome analysis. Because of these characteristics, the isolated *Arthrobacter* strains were targeted as a possible platform for exploring genomic features that may be related to microbial adaptation to drastically changed environments.

### Whole-genome sequencing of the isolated *Arthrobacter* strains

**Fig. 2:**
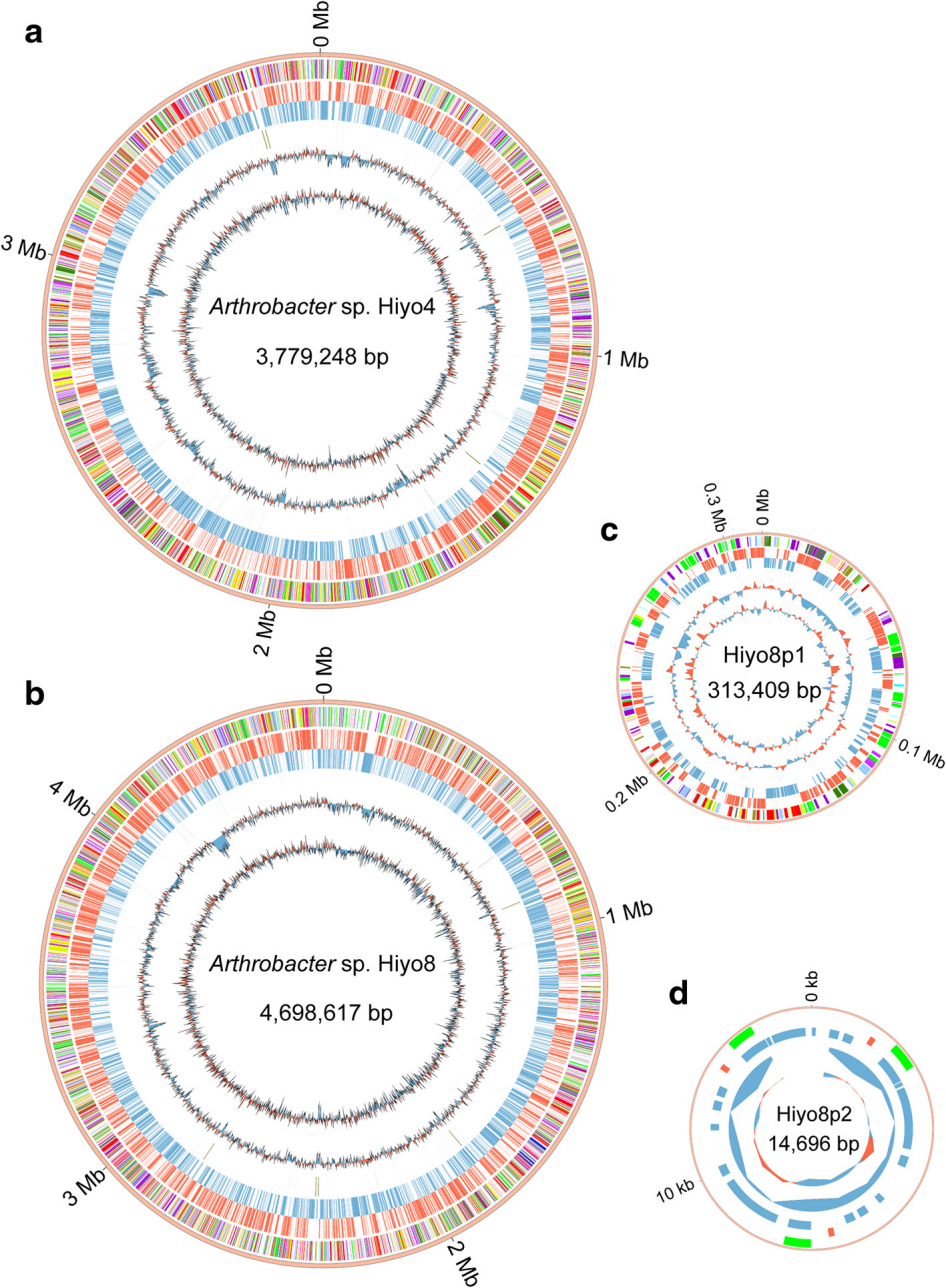
Circular diagrams of the chromosomes and plasmids of *Arthrobacter* sp. Hiyo4 and Hiyo8. Each concentric circle represents genomic data of *Arthrobacter* sp. **a** Hiyo4 and **b** Hiyo8 chromosomes and the Hiyo8 **c** p1 and **d** p2 plasmids. The outermost circle is the contig, the 2nd circle are the coding genes colored according to the functional categories of the eggNOG database (see additional file 2 for color coding), the 3rd and 4th circles are the coding genes on the leading (*red*) and lagging (*blue*) strands, respectively, the 5th circle are the rRNA (*brown*) and tRNA (*green*) genes, the 6th circle is the GC content (1-kb sliding window), and the innermost circle represents the GC skew (1-kb sliding window)

The whole-genome sequences of four *Arthrobacter* sp. strains were determined (Table 1). Assembly using the reads from PacBio RS II showed the complete genomes of two strains: Hiyo4 with one circular chromosome (3.8 Mbp), and Hiyo8 with one circular chromosome (4.7 Mbp) and two plasmids (0.3 Mbp and 15 kbp) (Fig. 2). Assembly using reads from GS FLX+ and Ion PGM System produced 38 and 630 scaffolds for two strains, Hiyo1 and Hiyo6, respectively.

**Table 1:** Whole-genome sequencing of the isolated *Arthrobacter* sp. strains

	Hiyo1	Hiyo6	Hiyo4	Hiyo8
Sequencing platform	GS FLX+ & Ion PGM	GS FLX+ & Ion PGM	PacBio RS II	PacBio RS II
Scaffolds	38	630	1	3
Contigs	1,685	2,450	1	3
Total genome size (bp)	5,543,883 ^a^	2,594,729 ^a^	3,779,248	4,698,617 ^a^
N50 (bp)	4,950	2,656	-	-
Coverage	20x	24x	79x	42x
GC content (%)	63.2	63.3	65.0	63.8
CDSs	5,292	3,767	5,120	7,041
rRNAs	2	3	12	15
tRNAs	51	33	50	53

^a^ Plasmid sequences were not excluded

The total genome sizes of the four strains ranged from 2.6 to 5.5 Mbp. Although the genome sizes of Hiyo4 and Hiyo8 were within the range of the previously reported genomes (Additional file 1), their CDS numbers were exceptionally large, possibly because of additional genes that facilitate adaptation to different environmental conditions. The functional categories of eggNOG were assigned to 61–66 % of the CDSs, and the GC content was 63–65 %, which is similar to that of the previously reported genomes (59–67 %) (Additional file 1).

### Phylogenetic analysis and comparative genomics

To reveal the phylogenetic relationships among the four strains Hiyo1, Hiyo4, Hiyo6, and Hiyo8, we constructed a maximum-likelihood phylogenetic tree of the *Arthrobacter* genus based on 16S rRNA gene sequences (Additional file 3). The tree reliably placed the four isolated strains within this genus. There was only one nucleotide base gap between the 16S rRNA sequences of Hiyo1 and Hiyo8, suggesting their close relationship. Hiyo4 and Hiyo6 were classified into different clades in the tree.

**Fig. 3:**
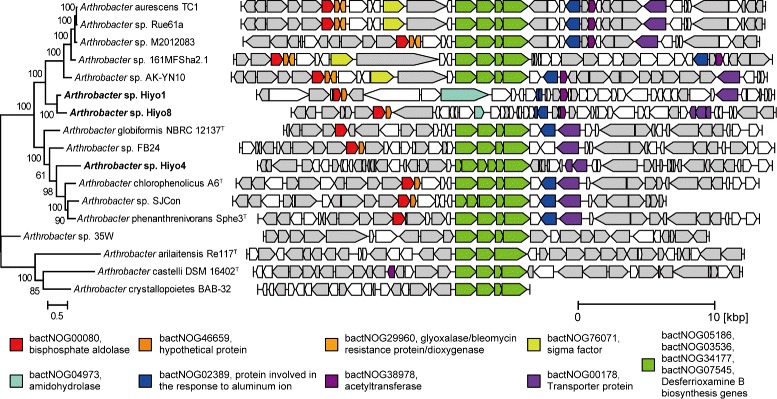
Syntenic map around siderophore-synthesis gene clusters in *Arthrobacter* genomes. Arrows represent genes whose lengths are proportional to the gene lengths. Desferrioxamine B biosynthesis genes are shown in green. Other conserved genes are shown in different colors according to their annotation. Other genes annotated using the eggNOG database are shown in gray. The phylogenetic tree was reconstructed on the basis of the set of 400 conserved bacterial marker genes with 1000 bootstrap replicates by the maximum-likelihood method

Subsequently, we conducted comparative genome analysis with 21 publicly available *Arthrobacter* genomes. The relative abundance of the CDSs assigned to each eggNOG functional category in each genome (Additional file 2) shows small difference among these *Arthrobacter* strains, i.e., their genomes have similar functional composition overall. The most striking difference between the *Arthrobacter* genomes isolated from the tsunami-affected soil and those isolated from other environments was that desferrioxamine B biosynthesis genes were independently lost in each of the former genomes. Within 14 publicly available, high-quality *Arthrobacter* genomes, the desferrioxamine B biosynthesis gene cluster and surrounding synteny structures were found to be highly conserved (Fig. 3). On the other hand, the desferrioxamine B biosynthesis gene cluster was entirely absent in the completed Hiyo1 and Hiyo8 genomes: the *desA* (pyridoxal-dependent decarboxylase) and *desB* (L-lysine 6-monooxygenase) genes of the cluster had nonsense mutations in the Hiyo4 genome, and neither the cluster nor the surrounding synteny structure was found in the Hiyo6 genome.

Desferrioxamine B is a member of the siderophores family of molecules, which are low-molecular-weight, iron-chelating compounds secreted by many microbes and plants for the uptake of iron [14–16, 49, 50]. The ability to use siderophores confers an ecological advantage when iron is limited [51]. Many *Arthrobacter* strains have a desferrioxamine B biosynthesis gene cluster, which is composed of four genes, named *desABCD*, for siderophore production [52, 53]. It should be noted that no iron-rich media were used during the isolation procedures.

**Fig. 4:**
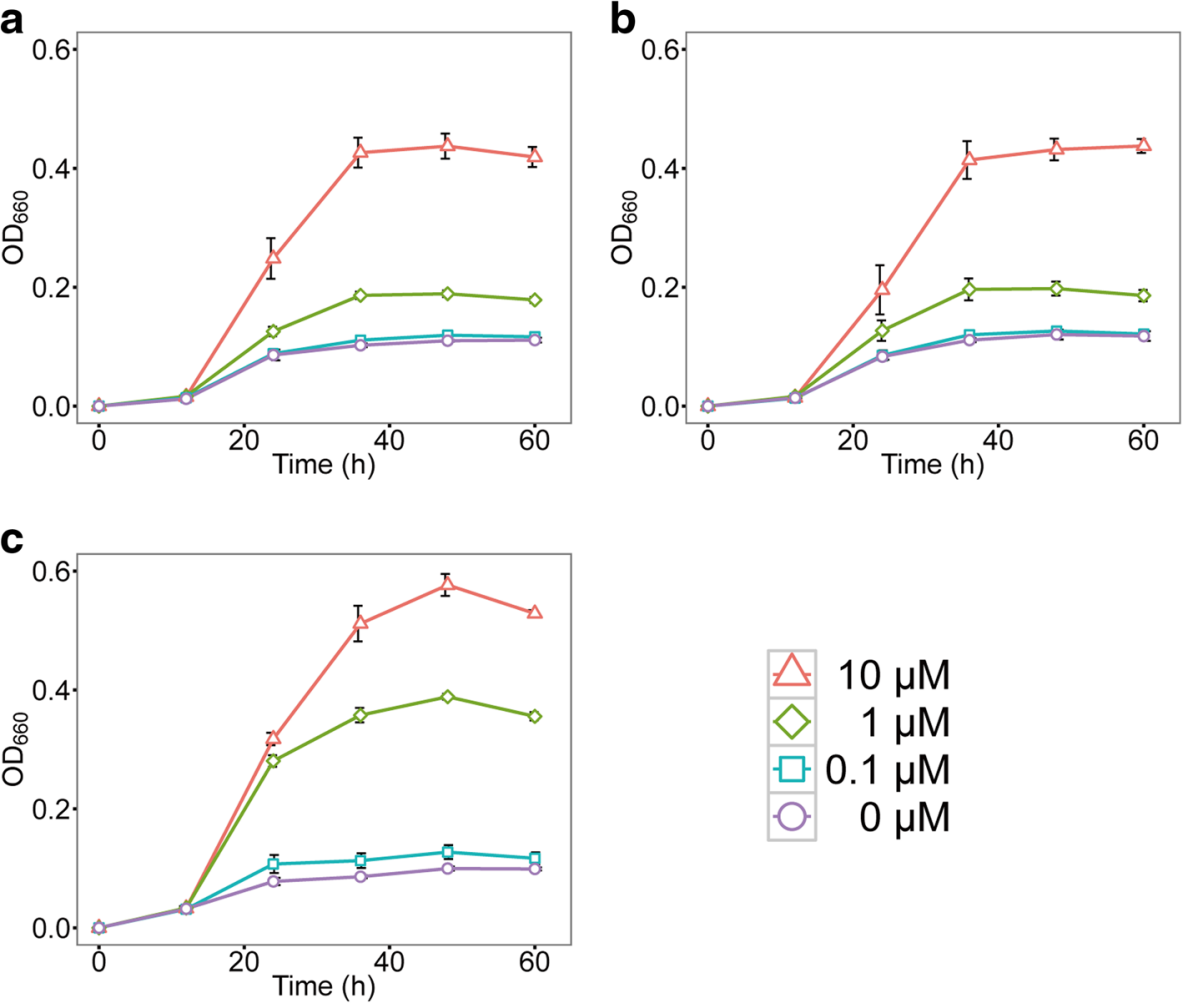
Growth curves of the *Arthrobacter* strains at different iron concentrations. Growth was measured as optical density values at 660 nm in modified MM9 medium containing different concentrations of iron (III): 0.0, 0.1, 1, and 10 *μ*M. Growth curves of *Arthrobacter* sp. Hiyo1 (**a**), *Arthrobacter* sp. Hiyo8 (**b**), and *A. phenanthrenivorans* Sphe3 (**c**) are shown. Growth curves of Hiyo4 and Hiyo6, which showed very weak growth in the modified MM9 medium, are displayed in Additional file 4

The independent losses of the siderophore-synthesis genes are not likely to have occurred by chance but likely because of natural selection. Thus, these *Arthrobacter* strains were assumed to have been under weak selection pressure for iron uptake and to be at a growth disadvantage under low iron concentrations. To evaluate the growth potentials of these strains under various iron concentrations, culture experiments in iron-controlled media were conducted (Fig. 4; Additional file 4). Two of the isolated strains (Hiyo1 and Hiyo8) required 10 *μ*M Fe^3+^ iron for rapid growth, whereas a control strain (*A. phenanthrenivorans* Sphe3) that has a desferrioxamine B biosynthesis gene cluster required 1 *μ*M Fe^3+^ iron (Fig. 4). Notably, under the 1 *μ*M Fe^3+^ iron concentrations, the maximum growth rates of Hiyo1 and Hiyo8 (1.08 ± 0.14 ×10^−2^ and 1.15 ± 0.32 ×10^−2^, respectively) was significantly smaller than that of Sphe3 (2.34 ± 0.20 ×10^−2^) (p-value <0.05, t-test with Bonferroni correction). Hiyo4 and Hiyo6 showed very weak growth even with 10 *μ*M Fe^3+^ iron, possibly because these two strains require additional nutrients for growth (Additional file 4 C and D).

Based on these results, we hypothesized that strains with *de novo* mutations in siderophore synthesis genes or those that originally lacked these genes would be selected under iron-enriched conditions. Notably, siderophore production by soil-living microbes has been reported to help various plants absorb iron (e.g., tomato, cucumber, barley, and corn) [54–56] and has been associated with N_2_ fixation (pigeon pea) [57]; therefore, these observed genomic changes in the bacterial communities might also relate to plant growth.

### Soil chemical analysis

To confirm that the tsunami-affected soil sample analyzed in this study did in fact have a high iron concentration and/or chemical characteristics that similar to those reported in previous studies on tsunami-affected soils, chemical analysis of the soil samples of the Hiyoriyama and Amamiya sites was conducted (Table 2). As expected, Hiyoriyama contained 13 times more iron than Amamiya, which is consistent with the observed losses of the siderophore-synthesis genes. Because Hiyoriyama was also found to be substantially rich in sulfate (e.g., SO$_{4}^{2-}$ levels were 169 times higher in Hiyoriyama than Amamiya), the iron was possibly provided in the form of iron-sulfur compounds (i.e., FeS, FeS_2_, Fe_2_S_3_), which are contained in seawater and sediment [58]. These sulfurs can be oxidized into sulfates via biological processes in the presence of electron acceptors [59], including nitrate (NO$_{3}^{-}$) [60–63]. We propose that the substantially smaller amount of nitrate in Hiyoriyama than Amamiya (>13-fold) may reflect this process.

**Table 2:** Chemical characteristics of the soil samples

		Hiyoriyama	Amamiya
pH	-	5.9	6.1
Electrical conductivity	dS/m	0.23	0.02
Total organic carbon	g/kg	3	2.4
Total nitrogen	g/kg	0.2	0.2
Ammonium nitrogen (NH_3_)	mg/kg	19.9	15.9
Nitrate nitrogen (NO$_{3}^{-}$)	mg/kg	6.2	84.5
Nitrite nitrogen (NO$_{2}^{-}$)	mg/kg	<0.05	<0.05
Effective phosphate (PO$_{4}^{3-}$)	mg/kg	12	40
Exchangeable K _+_	cmol(+)/kg	0.34	0.36
Exchangeable Ca ^2+^	cmol(+)/kg	6.19	4.32
Exchangeable Mg ^2+^	cmol(+)/kg	0.44	0.94
Exchangeable Na ^+^	cmol(+)/kg	0.42	0.10
Exchangeable Mn ^2+^	mg/kg	1.52	3.07
Available iron (Fe)	mg/kg	142	10.4
Cl ^−^	mg/kg	15.7	12.4
Sulfate (SO$_{4}^{2-}$)	mg/kg	379	2
Cd ^*a*^	mg/l	<0.001	<0.001
Cr(VI) ^*a*^	mg/l	<0.005	<0.005
Total mercury (Hg) ^*a*^	mg/l	<0.0005	<0.0005
Alkyl mercury (Hg) ^*a*^	mg/l	<0.0005	<0.0005
Pb ^*a*^	mg/l	<0.004	<0.004
As ^*a*^	mg/l	<0.001	0.001
B ^*a*^	mg/l	<0.1	<0.1
Cd ^*b*^	mg/kg	<15	<15
Cr(VI) ^*b*^	mg/kg	<25	<25
Hg ^*b*^	mg/kg	<1.5	<1.5
Pb ^*b*^	mg/kg	<15	<15
As ^*b*^	mg/kg	<15	<15
B ^*b*^	mg/kg	<400	<400
Cu ^*b*^	mg/kg	<10	<10
Zn ^*b*^	mg/kg	100	49
Ni ^*b*^	mg/kg	<30	<30

^a^Elution amount of chemicals by water

^b^Total amount of chemicals contained in the soil sample

Except for these chemicals, the characteristics of the two samples were similar overall, suggesting that the two soil samples share a similar geological origin. In particular, the absence of heavy metals such as Pb, Hg, and Cu in Hiyoriyama might indicate that the soil was not completely covered or replaced with marine sediments. In addition, the similarities in electrical conductivity and Na^+^ and Cl^-^ content between samples can be attributed to the effects of rain; in the case of the 2004 Indian Ocean tsunami, water-soluble salts derived from the tsunami were strongly reduced after a rainy season in a coastal area in Thailand [64].

### Shotgun metagenome sequencing

To investigate differences in the taxonomic compositions and protein-coding gene abundance between the two samples, shotgun metagenome sequencing was conducted (Table 3). After quality control, 822,865 and 961,221 reads were obtained from the Hiyoriyama and Amamiya samples, respectively.

**Table 3:** General features of the metagenome sequences

	Hiyoriyama	Amamiya
Raw sequence reads	1,091,366	1,177,491
After quality control	822,865 (75.40 %)	961,221 (81.60 %)
CDSs	1,170,916	1,323,575
16S rRNAs	628	633
Taxonomically classified reads	114,838 (13.96 %)	112,459 (11.70 %)
- Bacteria	113,696 (99.01 %)	111,326 (98.99 %)
- Archaea	933 (0.81 %)	707 (0.63 %)
- Viruses	209 (0.18 %)	426 (0.38 %)

**Fig. 5:**
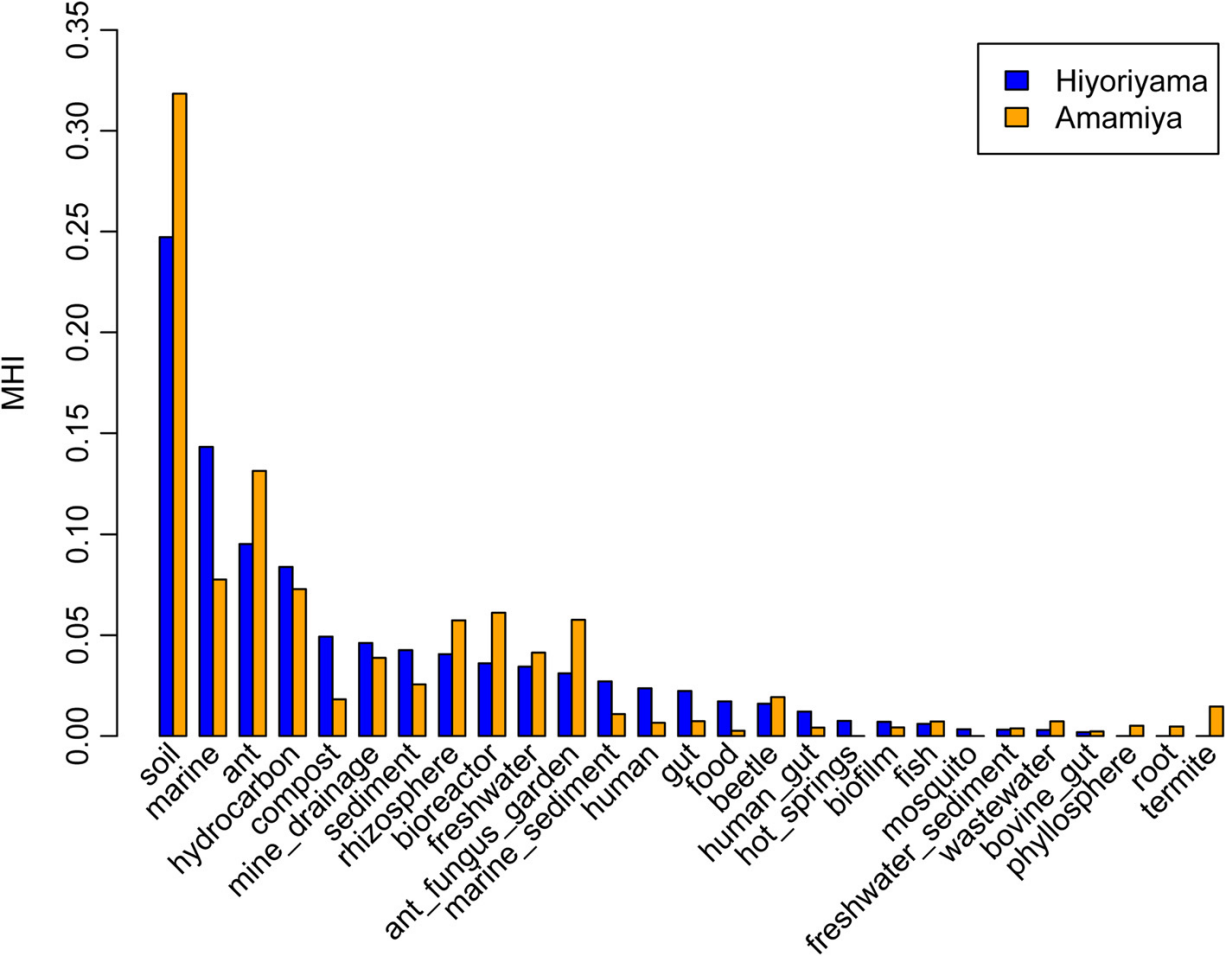
Estimated habitats of microbes at each site. Microbial habitability index (MHI) scores were calculated using the top-hit sequences of blastn searches against MetaMetaDB, where queries were all 16S rRNA gene sequences extracted from the shotgun metagenome sequences. Blue and orange bars represent Hiyoriyama and Amamiya, respectively

Using Kraken [33], 114,838 (14.0 %) and 112,459 (11.7 %) shotgun reads from Hiyoriyama and Amamiya were taxonomically classified, respectively. Almost all reads were assigned to Bacteria (99.01 and 98.99 %), whereas few reads were assigned to Archaea (0.81 and 0.63 %) and Viruses (0.18 and 0.38 %). The microbial composition of abundant genera is shown in Additional file 5. The most abundant genus in both samples was *Burkholderia* (4.85 and 7.20 %), followed by *Bradyrhizobium* (4.66 and 6.30 %), *Rhodopseudomonas* (3.27 and 3.46 %), and *Pseudomonas* (2.98 and 3.13 %). The similar composition of the major taxonomic groups reflects the similar overall chemical characteristics between the two soil samples. In addition, we estimated the typical habitats of the contained microbes by querying the extracted 16S rRNA genes against MetaMetaDB [37], a database that links 16S rRNA gene sequences to environments based on comprehensive analysis of published metagenomic and amplicon-sequencing datasets. The estimated habitats quantified as MHI values [37] showed that the top habitat was soil in both communities; however, the marine habitat was estimated to be modestly more abundant in Hiyoriyama, whereas the soil habitat was more abundant in Amamiya, as expected (Fig. 5).

**Fig. 6:**
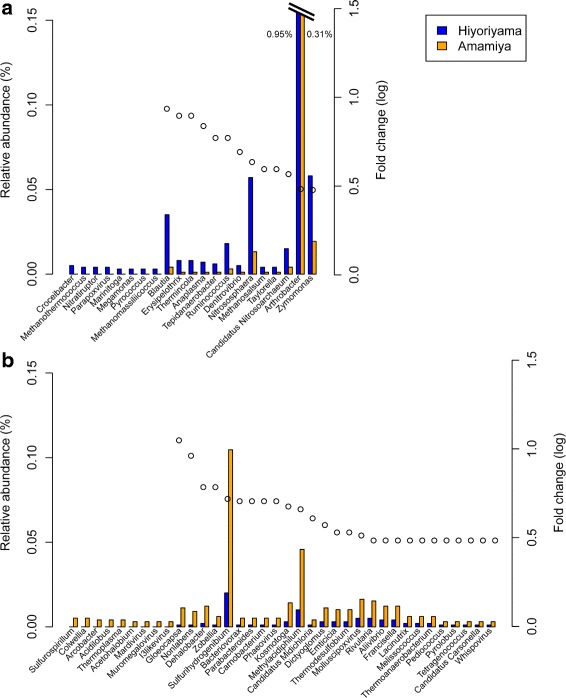
Microbial genera with a substantial difference in abundance between the two sites. Genera whose relative abundance values differed by more than three times between the two sites and represented more than 0.003 % of the total abundance in either of the two sites are displayed for genera overrepresented in **a** Hiyoriyama and **b** Amamiya. Blue and orange bars represent the relative abundance in Hiyoriyama and Amamiya, respectively. Dots represent the ratios of larger abundance values divided by smaller values, if the smaller value was not zero

Figure 6 displays the genera whose relative abundance substantially differed between the two samples, including only those whose abundance in one sample was more than three times greater than that in the other. Notably, *Arthrobacter* was the only genus that was both abundant in and differed substantialy between the two samples. Considering the fact that *Arthrobacter* was the genus cultivated in both the R2A and ZoBell media, we propose that this genus likely shows a greater potential for adaptation to tsunami-affected soils. The other genera that were more abundant in Hiyoriyama included *Erysipelothrix*, where all reads were assigned to a single species, *Erysipelothrix rhusiopathiae* [65], which is known to cause erysipelas, a bacterial skin infection, in animals [66]. Although previous culture-based studies reported several pathogen species (*Mycobacterium elephantis*, *Massilia timonae*, *Vibrio ichthyoenteri*, *V. natriegens*, and *V. fluvialis*) in sludge derived from tsunami-affected soil in Tohoku [13, 67], these species were not detected in the present dataset. It may also be notable that the genera detected only in Hiyoriyama included typical marine-living groups such as *Croceibacter* [68], *Marinitoga* [69], and *Pyrococcus* [70–72], implying that the tsunami facilitated microbial immigration.

**Fig. 7:**
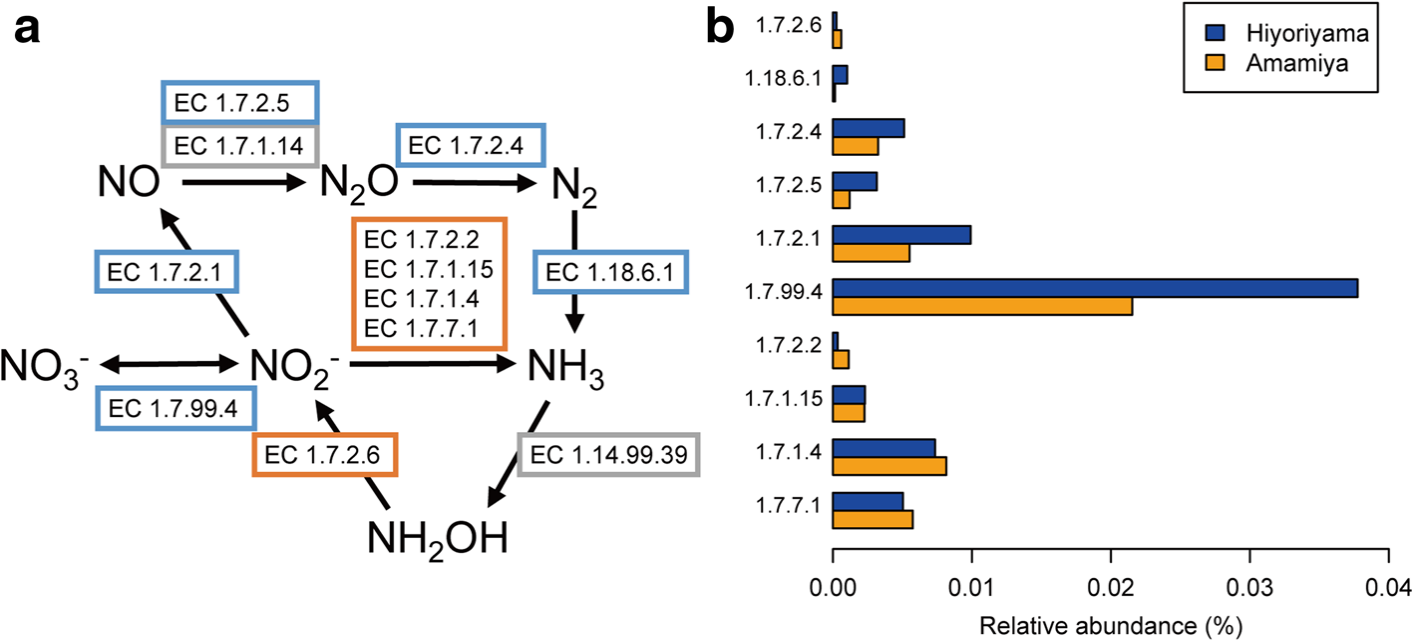
Relative abundance of genes related to nitrogen metabolism. **a** A pathway map of nitrogen metabolism genes with Enzyme Commission numbers. Blue and orange rectangles represent genes that were found to be more abundant in Hiyoriyama and Amamiya, respectively. Gray rectangles represent genes not found in either sample. **b** A bar plot of the relative abundance for each gene represented by an Enzyme Commission number

We annotated the CDSs and investigated the relative abundance of nitrogen cycle-related genes, because the taxonomic analysis identified genera known to metabolize inorganic nitrogens, such as *Bradyrhizobium*, *Azospirillum*, *Frankia*, *Mesorhizobium*, *Rhizobium*, and *Sinorhizobium* (Additional file 5), and the chemical analysis revealed differences in the amount of nitrogen compounds (Table 2). The abundance of functional genes showed that genes related to denitrification and nitrogen fixation were more abundant in Hiyoriyama and genes related to nitrite reduction were more abundant in Amamiya (Fig. 7). In addition to the oxidization of iron-sulfur compounds, this dominance of denitrification-related genes at Hiyoriyama may be another cause of the relatively small amount of nitrate observed in Hiyoriyama (Table 2), which might affect terrestrial vegetation indirectly.

We also investigated the abundance of siderophore-synthesis genes in the shotgun metagenome data, but only three and four reads of genes that are involved in this process (bactNOG07545, bactNOG14638, bactNOG30540) were detected in Hiyoriyama and Amamiya, respectively, which is not a sufficient sample size for statistical analysis. Differences in sulfur metabolism genes were not as large as those of nitrogen metabolism genes. A substantial difference was observed in the numbers of cation transporter genes, where 127 and 59 monovalent cation/H^+^ antiporter subunits, and 96 and 16 Na^+^/Ca^2+^ antiporter family proteins (bactNOG00892) were detected in Hiyoriyama and Amamiya, respectively. These genes may have facilitated salt tolerance in the tsunami-affected soil, because cation transporters are known to function in bacterial salt tolerance [73, 74].

## Conclusion

In this study, we isolated four *Arthrobacter* strains from a soil sample affected by the Tohoku tsunami and determined their whole-genome sequences. Independent losses of siderophore-synthesis genes were suggested in these genomes, which was consistent with the rich iron content detected in the tsunami-affected soil sample and the weak cultivability of the isolated strains in iron-limited media, although further experimental analysis will be needed to conclude it. The chemical and metagenomic analyses indicated that the tsunami-affected sample was largely similar to the unaffected sample, although some notable differences were observed regarding nitrogen metabolism and taxonomic composition. It should be noted that we cannot conclusively determine whether the isolated *Arthrobacter* strains were brought into the sampled area from sea and then adapted to soil, or were originally in the soil and survived under the tsunami-affected conditions. In either case, it also remains undetermined whether the siderophore-synthesis genes were mutated after the tsunami or the strains that originally lost these genes were simply favored and selected in the tsunami-affected soil.

The Pacific coast of Tohoku, Japan has been flooded by tsunamis many times in history (more than 11 tsunamis were triggered in the last 200 years, according to [75]). Because a tsunami should affect the soil and its microbial communities in diverse manners, we envision that further comprehensive analyses on microbial ecology and evolution after a tsunami will be necessary to develop a deeper understanding of the recovery processes of terrestrial microbial ecosystems.

## Additional files

Additional file 1
**Arthrobacter dataset for comparative genome analysis.** (PDF 66 kb)

Additional file 2
**Relative abundance of functional gene categories in the** ***Arthrobacter*** **genomes.** The relative abundance of CDSs assigned to each eggNOG functional category is plotted for each *Arthrobacter* genome. (PDF 166 kb)

Additional file 3
**Phylogenetic tree of** ***Arthrobacter*** **genus.** The phylogenetic tree was reconstructed using the maximum-likelihood method based on 16S rRNA sequences with *Streptomyces coelicoflavus* NBRC 15399^T^ as an outgroup. Numbers adjacent to branch points are bootstrap percentages (1000 replicates). Symbols represent the available circular genomes (circle), available draft genomes (triangle), those isolated in this study (red), and those isolated in previous studies (blue). (TIF 920 kb)

Additional file 4
**Growth curves of the** ***Arthrobacter*** **strains at different iron concentrations.** Growth was measured as optical density values at 660 nm in modified MM9 medium containing different concentrations of iron (III): 0.0, 0.1, 1, and 10 *μ*M. Growth curves of *Arthrobacter* sp. Hiyo1 (**A**), Hiyo8 (**B**), Hiyo4 (**C**), Hiyo6 (**D**), and *A. phenanthrenivorans* Sphe3 (**E**) were measured. (TIF 380 kb)

Additional file 5
**Abundant microbial genera determined in metagenome shotgun sequencing.** The 30 most abundant microbial genera at Hiyoriyama and their relative abundance at both sites are displayed. Blue and orange bars represent Hiyoriyama and Amamiya, respectively. (TIF 567 kb)
