# Supplementary material for: Genomic and metagenomic analysis of microbes in a soil environment affected by the 2011 Great East Japan Earthquake tsunami
Source: BMC Genomics. 2016 Jan 14;17:53. doi: 10.1186/s12864-016-2380-4 (PMC4712596; doi:10.1186/s12864-016-2380-4)
Supplement: Additional file 1 — Arthrobacter dataset for comparative genome analysis. (PDF 66 kb) [file 12864_2016_2380_MOESM1_ESM.pdf]

Additional file 1: *Arthrobacter* dataset for comparative genome analysis.

**Species Name**	**Strain name**	**NCBI taxonomy ID**	**Isolated environment**	**Status**	**Type strain**	**Total genome size (bp)**	**Number of scaffolds**	**GC content (%)**	**CDSs**	**rRNA**	**tRNA**
Arthrobacter arilaitensis	Re117	861360	Cheese	Complete	T	3,918,192	3	59.3	3,736	18	64
Arthrobacter aurescens	TC1	290340	Soil	Complete	F	5,226,648	3	62.4	4,819	18	54
Arthrobacter castelli	DSM 16402	1121019	Mural painting	Draft	T	4,582,606	52	63.6	4,453	4	46
Arthrobacter chlorophenolicus	A6	452863	Soil	Complete	T	4,980,870	3	66	4,645	15	85
Arthrobacter crystallopoietes	BAB-32	1246476	Soil	Draft	F	4,348,607	347	66.7	4,387	4	51
Arthrobacter gangotriensis	Lz1y	1276920	Soil	Draft	T	4,319,900	20	63	4,029	4	58
Arthrobacter globiformis	NBRC 12137	1077972	Soil	Draft	T	4,954,410	125	66.2	4,544	3	50
Arthrobacter phenanthrenivorans	Sphe3	930171	Soil	Complete	T	4,535,320	3	65.4	4,246	12	50
Arthrobacter sp.	131MFCol6.1	1157944	Rhizosphere	Draft	F	4,432,383	29	67.2	3,968	5	50
Arthrobacter sp.	135MFCol5.1	1158050	Rhizosphere	Draft	F	4,453,574	37	66.1	4,083	7	50
Arthrobacter sp.	161MFSha2.1	1151118	Rhizosphere	Draft	F	4,572,124	44	63.1	4,295	6	54
Arthrobacter sp.	162MFSha1.1	1151119	Rhizosphere	Draft	F	4,399,171	55	66.1	4,107	6	51
Arthrobacter sp.	35W	1132441	Lake	Draft	F	4,652,932	6	66.8	4,103	12	54
Arthrobacter sp.	AK-YN10	1349820	Soil	Draft	F	4,839,751	107	63.3	4,614	2	53
Arthrobacter sp.	CAL618	1055770	Human	Draft	F	3,654,388	288	63.2	3,642	18	47
Arthrobacter sp.	FB24	290399	Soil	Complete	F	5,070,478	4	65.4	4,624	15	51

Arthrobacter sp.	Hiyo1	1588020	(This study)	Draft	F	5,543,883	38	63.2	5,292	2	51
Arthrobacter sp.	Hiyo4	1588021	(This study)	Complete	F	3,790,568	1	65	5,120	12	50
Arthrobacter sp.	Hiyo6	1588022	(This study)	Draft	F	2,594,729	630	63.3	3,767	3	33
Arthrobacter sp.	Hiyo8	1588023	(This study)	Complete	F	4,698,617	3	63.8	7,041	15	53
Arthrobacter sp.	M2012083	1197706	Soil	Draft	F	4,629,172	67	62	4,304	3	54
Arthrobacter sp.	Rue61a	1118963	Wastewater	Complete	F	5,081,038	3	62.2	4,723	18	53
Arthrobacter sp.	SJCon	683150	Soil	Draft	F	4,389,620	142	66.2	4,635	3	50
Arthrobacter sp.	TB 23	494419	Sponge	Draft	F	3,542,308	126	63.3	3,405	15	46
Arthrobacter sp.	TB 26	494420	Human	Draft	F	4,324,615	556	66.4	4,451	19	50
