# Supplementary material for: Genomic and metagenomic analysis of microbes in a soil environment affected by the 2011 Great East Japan Earthquake tsunami
Source: BMC Genomics. 2016 Jan 14;17:53. doi: 10.1186/s12864-016-2380-4 (PMC4712596; doi:10.1186/s12864-016-2380-4)
Supplement: Additional file 2 — Relative abundance of functional gene categories in the Arthrobacter genomes. The relative abundance of CDSs assigned to each eggNOG functional category is plotted for each Arthrobacter genome. (PDF 166 kb) [file 12864_2016_2380_MOESM2_ESM.pdf]

Additional file 2: Relative abundance of functional gene categories in the *Arthrobacter* genomes.

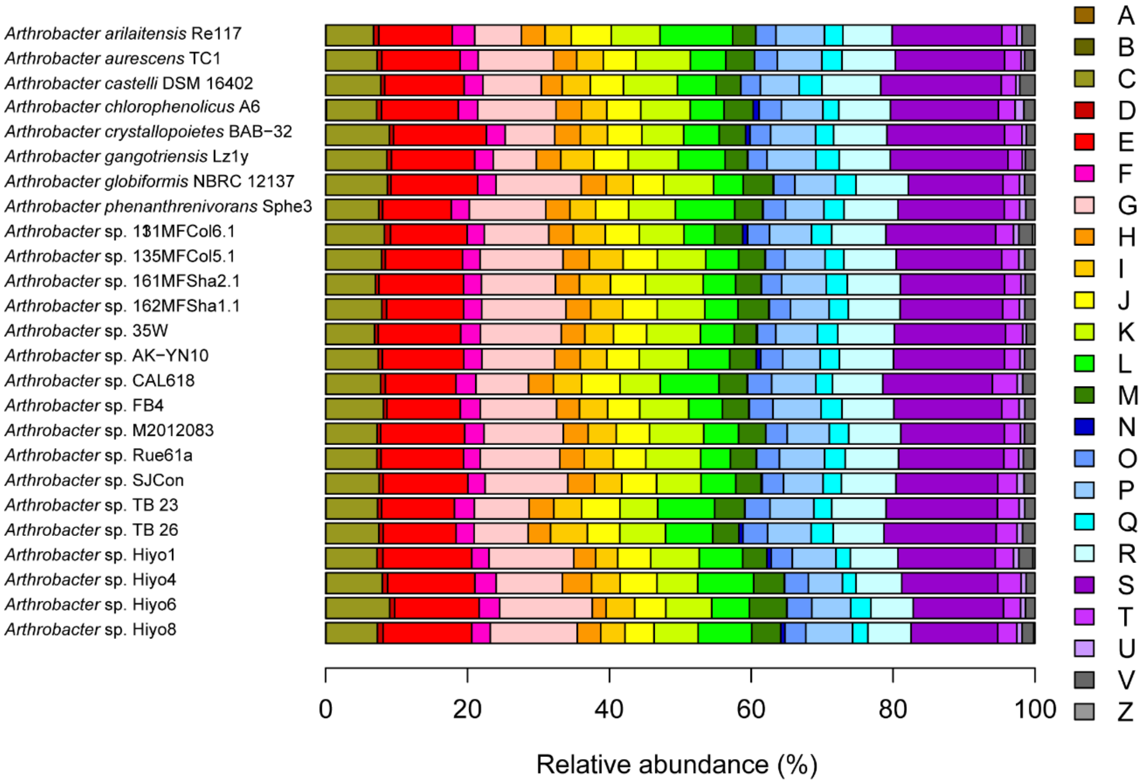

Relative abundance of CDSs assigned to each eggNOG functional category is plotted for each *Arthrobacter* genome. The eggNOG functional categories are as follows: A, RNA processing and modification; B, chromatin structure and dynamics; C, energy production and conversion; D, cell cycle control, cell division and chromosome partitioning; E, amino acid transport and metabolism; F, nucleotide transport and metabolism; G, carbohydrate transport and metabolism; H, coenzyme transport and metabolism; I, lipid transport and metabolism; J, translation; K, transcription; L, replication; M, cell wall/membrane/envelope biogenesis; N, cell motility; O, posttranslational modification, protein turnover, chaperones; P, inorganic ion transport and metabolism; Q, secondary metabolites biosynthesis, transport and catabolism; R, general function prediction only; S, function unknown; T, signal transduction mechanisms; U, intracellular trafficking and secretion; V, defense mechanisms; W, extracellular structures; Y, nuclear structure; and Z, cytoskeleton.
