# Supplementary figures and images for: Genomic and metagenomic analysis of microbes in a soil environment affected by the 2011 Great East Japan Earthquake tsunami
Source: BMC Genomics. 2016 Jan 14;17:53. doi: 10.1186/s12864-016-2380-4 (PMC4712596; doi:10.1186/s12864-016-2380-4)

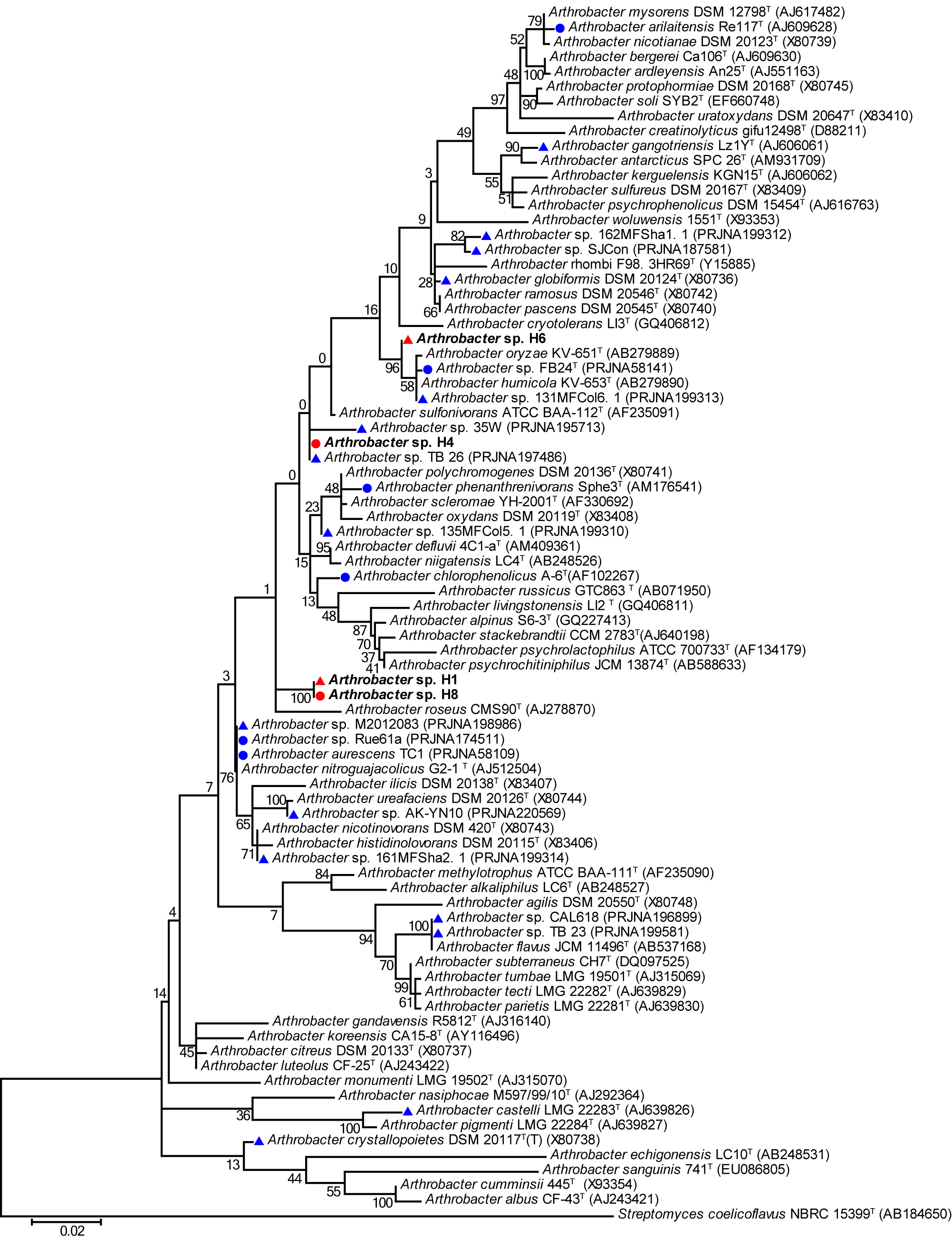

Supplement: Additional file 3 — Phylogenetic tree of Arthrobacter genus. The phylogenetic tree was reconstructed using the maximum-likelihood method based on 16S rRNA sequences with Streptomyces coelicoflavus NBRC 15399T as an outgroup. Numbers adjacent to branch points are bootstrap percentages (1000 replicates). Symbols represent the available circular genomes (circle), available draft genomes (triangle), those isolated in this study (red), and those isolated in previous studies (blue). (TIF 920 kb) [file 12864_2016_2380_MOESM3_ESM.tif]

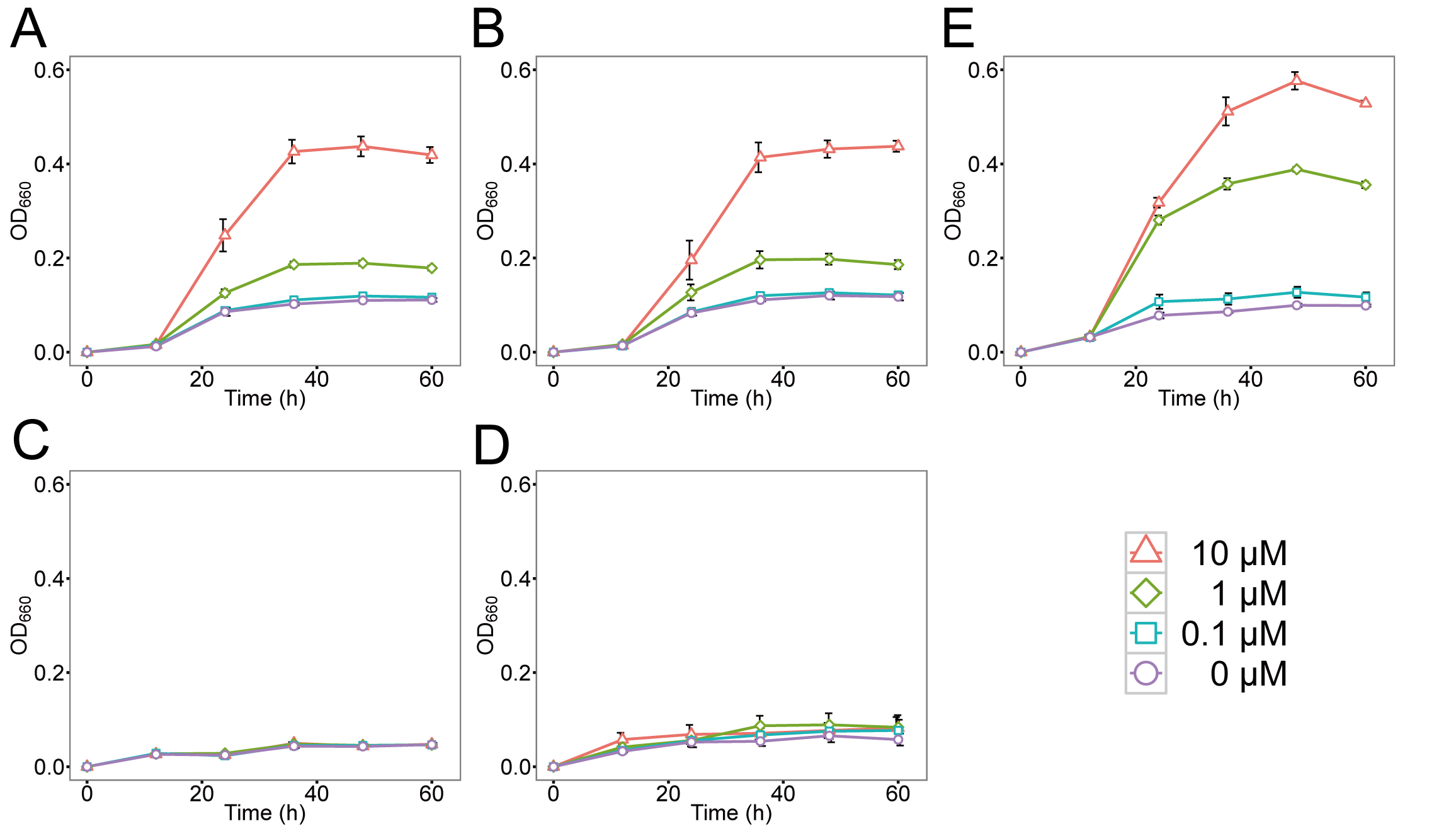

Supplement: Additional file 4 — Growth curves of the Arthrobacter strains at different iron concentrations. Growth was measured as optical density values at 660 nm in modified MM9 medium containing different concentrations of iron (III): 0.0, 0.1, 1, and 10 μM. Growth curves of Arthrobacter sp. Hiyo1 (A), Hiyo8 (B), Hiyo4 (C), Hiyo6 (D), and A. phenanthrenivorans Sphe3 (E) were measured. (TIF 380 kb) [file 12864_2016_2380_MOESM4_ESM.tif]

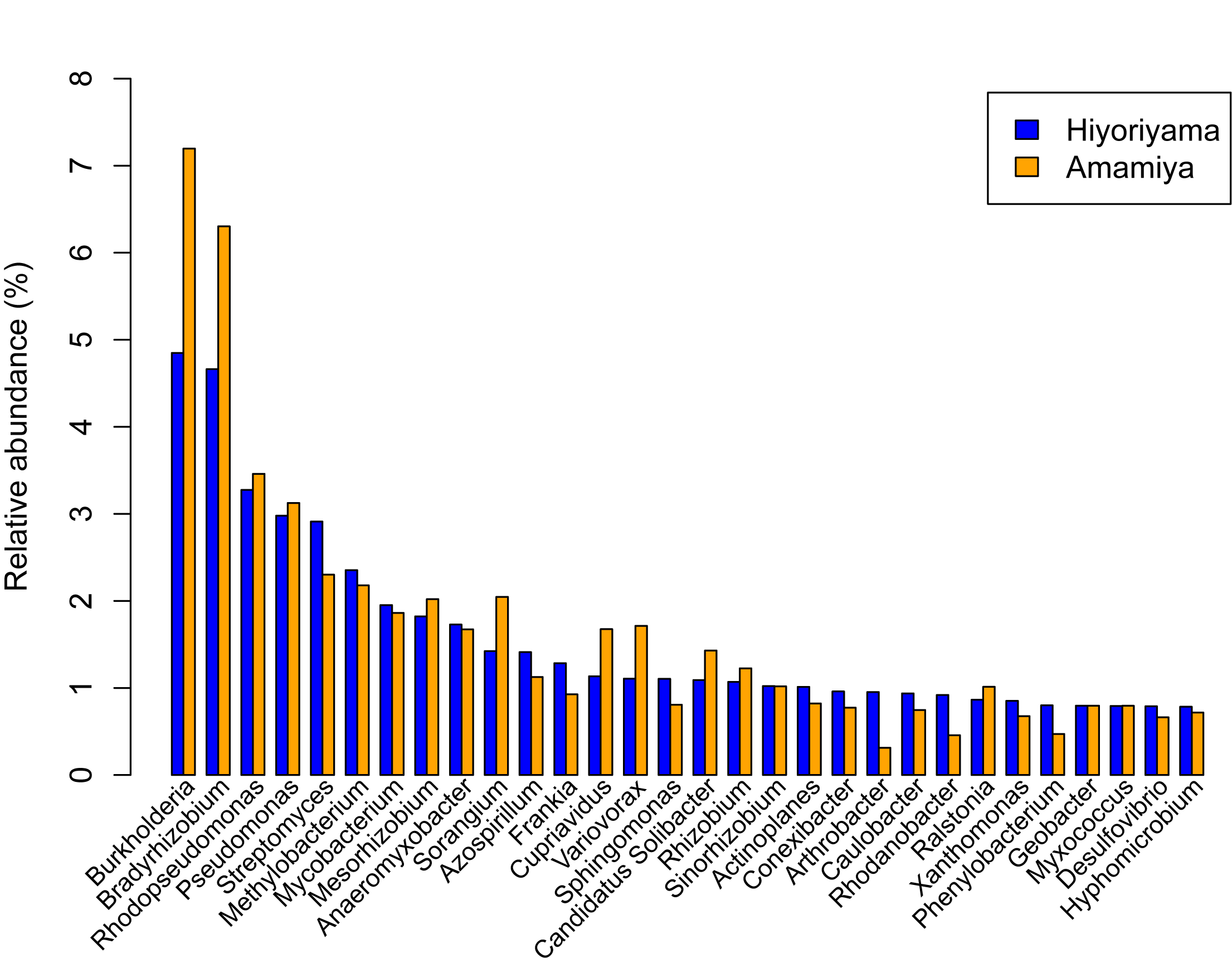

Supplement: Additional file 5 — Abundant microbial genera determined in metagenome shotgun sequencing. The 30 most abundant microbial genera at Hiyoriyama and their relative abundance at both sites are displayed. Blue and orange bars represent Hiyoriyama and Amamiya, respectively. (TIF 567 kb) [file 12864_2016_2380_MOESM5_ESM.tif]
